# Collagen of Extracellular Matrix from Marine Invertebrates and Its Medical Applications

**DOI:** 10.3390/md17020118

**Published:** 2019-02-14

**Authors:** M. Azizur Rahman

**Affiliations:** 1 Department of Chemical & Physical Sciences, University of Toronto, Mississauga, ON L5L 1C6, Canada; aziz@climatechangeresearch.ca or mazizur.rahman@utoronto.ca; Tel.: +1-647-892-4221; 2 Center for Climate Change Research, Toronto, ON M4P 1J4, Canada

**Keywords:** collagen, chitin, corals, extracellular matrix, marine invertebrates, marine proteins

## Abstract

The extraction and purification of collagen are of great interest due to its biological function and medicinal applications. Although marine invertebrates are abundant in the animal kingdom, our knowledge of their extracellular matrix (ECM), which mainly contains collagen, is lacking. The functions of collagen isolated from marine invertebrates remain an untouched source of the proteinaceous component in the development of groundbreaking pharmaceuticals. This review will give an overview of currently used collagens and their future applications, as well as the methodological issues of collagens from marine invertebrates for potential drug discovery.

## 1. Introduction

Collagen is one of the most abundant proteins in the extracellular matrix of animal bodies. This protein is the main fibrous, structural protein and supports the formation of all joints in the body. Supplementing collagen is an important way to keep our body healthy. Nowadays, collagen-based biomedical materials are used for the treatment of many human diseases (e.g., bone tissue regeneration). The challenge currently facing scientists is to find a suitable source of collagen, and the extraction and purification of collagen, which would be appropriate for applying to medical applications. 

**Figure 1:**
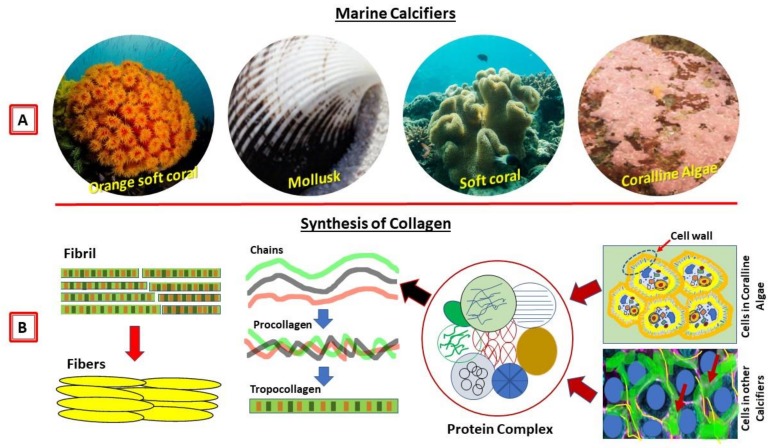
Marine calcifiers and their collagens. (**A**) Examples of marine calcifiers/invertebrates. (**B**) A model image on the biological synthesis of collagens from the marine invertebrates and crustose coralline algae.

There is a huge source of collagen from marine organisms, and recent research has demonstrated that the marine source is the most convenient and safest way to obtain it, with invertebrates and crustose coralline algae [1,2,3,4,5,6,7,8,9] being the most abundant and potential sources (see Figure 1A for examples). A marine source also has lots of advantages over land animals such as being environmentally friendly, having a high quantity of collagen, having biological toxins that are almost negligible, having better absorption due to low molecular weight, having a minimal inflammatory response, having less religious and ethical constraints, being metabolically compatible, and having few regulatory and quality control problems.

In this review, I included crustose coralline algae (CCA) because they have similar characteristics of proteinaceous components and mineralization processes like calcifying marine invertebrates. CCA are rock-hard calcareous with two key functional roles in coral reef ecosystems: (1) reef calcification and cementation and (2) inducing the larval settlement of many benthic organisms. CCA contain calcium carbonate with hard skeletons and minerals (e.g., calcite) similar to coral skeletons. In addition, CCA have a high content of organic matrix skeletal proteins, including chitin and collagen [5,9]. CCA are abundant and are found in marine waters all over the world. I therefore introduce these abundant marine sources with the invertebrates presented in this review, which might have a high potential for the extraction of collagens, and moreover use for medical applications.

Invertebrates make up almost 95% of the animal kingdom, but our knowledge of their extracellular matrices, in particular, the polymer collagen is very weak. The information on the biology of collagen within the extracellular matrix is scanty. A large number of marine invertebrates produce polysaccharides and extracellular matrices [10,11,12,13,14] within their connective tissues, and their molecular structures and functions are similar to humans [15,16]. Moreover, polysaccharides extracted from marine calcifiers that contain extracellular matrices have an enormous assortment of structures (Figure 1B), and they can be considered an extraordinary source of biochemical variety. We therefore discuss the studies of collagens of invertebrates (including related marine calcifiers) and their plausible medical application.

Treatment of bone defects such as replacing tissue or regeneration requires biomaterials with similar mechanical integrity to natural bone, which can adapt and contribute to the tissue growth processes. From an applicable biomaterials point of view, the mineralized extracellular matrix of collagen in marine invertebrate structures has a vast richness for tissue engineering [17,18]. The skeletons in marine invertebrates are classic bio-resources that have tailored architectures to give structural support, and their functions are feasible for human tissue regeneration and repair. Marine calcifiers, for example, coralline, sea urchin, and coral, have interconnected porous structures that are enriched with bioactive elements and medical materials that could be used for tissue engineering and drug design applications [5,11,12,19,20,21,22,23]. The main purpose of this review is to provide an overview of currently used collagens from marine invertebrates and related calcifying organisms, and their medicinal potential, as well as the technical issues in purifying collagen from them.

## 2. Current State of Collagen Research and its Medical Application

**Figure 2:**
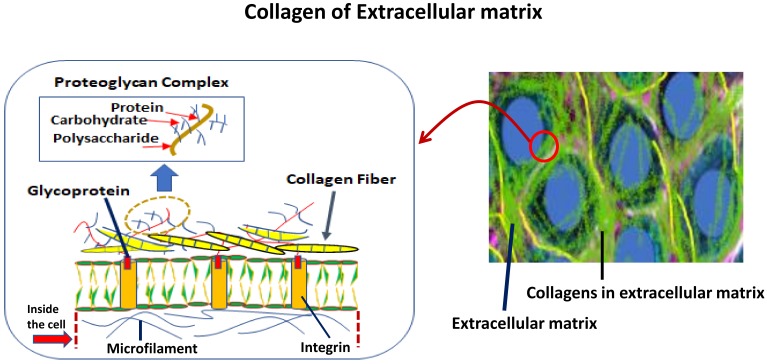
Collagen of extracellular matrix and its biology in invertebrates. The right panel shows a model of cells. The left panel shows the structural components of the extracellular matrix, which are involved in the formation of collagen in marine invertebrates.

**Figure 3:**
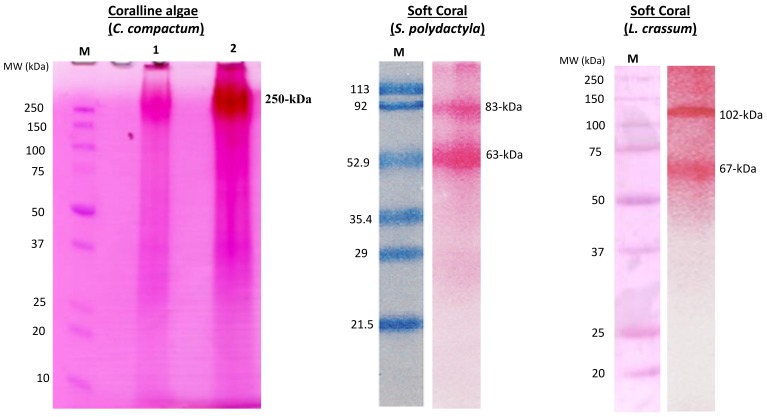
Collagen associated glycoproteins in marine calcifiers. Coralline red algae: Sodium dodecyl sulfate-polyacrylamide gel electrophoresis (SDS-PAGE) with a periodic acid-schiff (PAS) staining to detect glycoprotein in the extracellular matrix of *Clathromorphum compactum*. M, protein ladder. Lane 1 and 2, high molecular weight (250 kDa) of a glycoprotein. Soft coral (*Sinularia polydactyla*): SDS-PAGE with a PAS staining to detect glycoprotein in the extracellular matrix of *S. polydactyla*. M, protein ladder. Two glycoproteins (83 and 63 kDa) were identified in this species. Soft Coral (*Lobophytum crassum*): SDS-PAGE with a PAS staining. The PAS staining to detect glycoprotein in the extracellular matrix of *L. crassum*. M, protein ladder. Two glycoproteins (102 and 67 kDa) were identified in this soft coral species. The Precision Plus SDS-PAGE protein ladder (Bio-Rad) was used for the electrophoresis analysis of all above-mentioned glycoproteins. The glycoproteins presented here were reproduced from Rahman [5] for the coralline red algae and Rahman et al. [25] for the two soft corals (*S. polydactyla*, *L. crassum*).

There has been significant progress in the research of marine natural products in the purpose of medical application nowadays. Marine invertebrates are the main source of this purpose; however, finding collagen for the treatment of bone-related diseases is not well established yet. Many research groups have been studying collagen in some marine calcifier tissues with a focus on structure and functional relationships [1,2,3,4,5,6,7,8,9,10]. The biology of the extracellular matrix (Figure 2), particularly of collagen in invertebrates is essential to understanding the continuing research in the field of marine natural products. One of the key components of the structure of the collagen is a glycoprotein (see Figure 2, left panel), and many organisms in the CCA and invertebrate such as corals (especially, soft corals), coralline algae, and jewelry corals [5,24,25,26] have already demonstrated this key molecule (see Figure 3 for some examples). Helman et al. [24] reported collagen production in the ECM of both soft (*Xenia elongata*) and hard (*Montipora digitata*) corals. They clearly demonstrated the presence of glycoprotein in the ECM of corals, which means the presence of collagen must exist if the glycoprotein is present in the species. This is an indicator for species that contain collagen molecules.

To date, collagen has been identified in corals, sponges, sea urchin, salmon, jellyfish, mollusk, and coralline red algae [2,3,4,5,7,8,9,10,20,27,28,29,30,31,32,33], among others. Most of these organisms have also been applied for use in tissue engineering [34]. Collagen from marine invertebrates and related calcifiers have been discussed in numerous review papers [5,6,31,35,36,37,38,39,40,41,42] where the authors highlighted details regarding the structure and application of the collagen of this abundant marine source. It is a great possibility to use the huge source of marine invertebrates for extracting and purifying collagen, not only for the medical application and bone-related disease but also for use in cosmetics and anti-aging [43,44,45,46,47,48].

**Figure 4:**
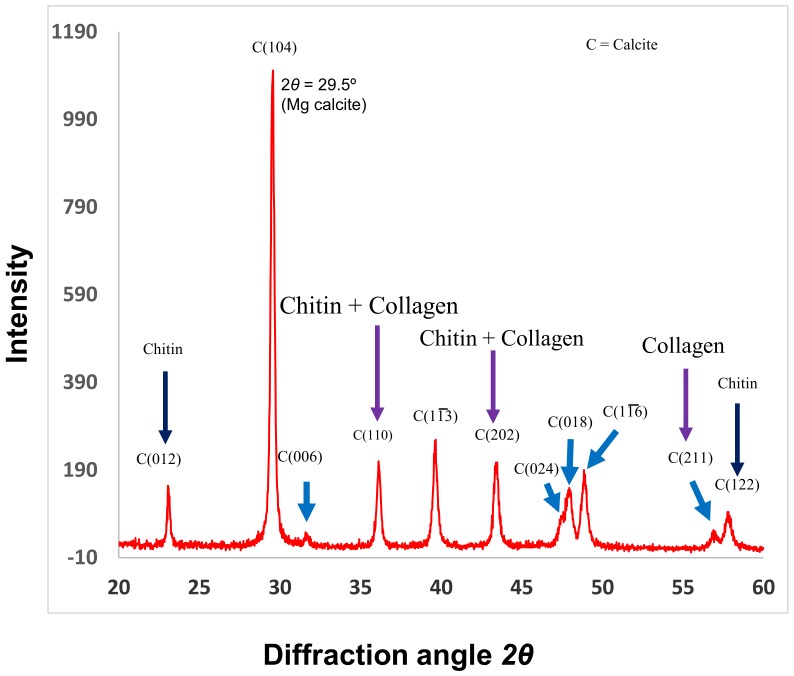
X-ray diffraction (XRD) analysis of *C. compactum*. The 2*θ* scan identifies the mineral form of CaCO_3_ crystal planes, which were nucleated by chitin and collagen matrices. Purple arrows show the collagen bands. Reproduced with permission from Rahman and Halfar [9].

**Figure 5:**
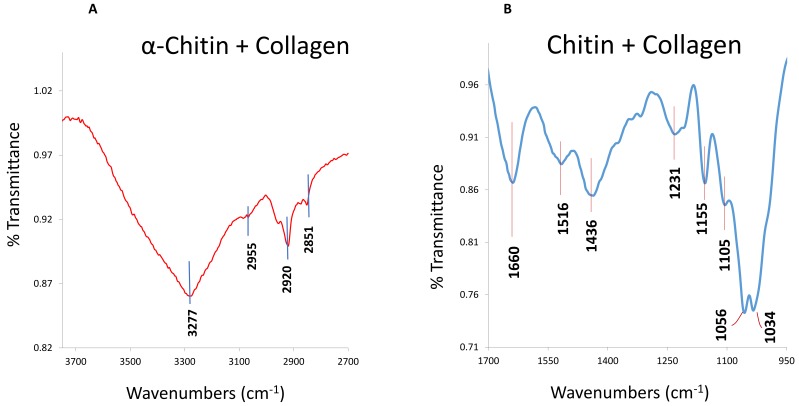
Infrared (IR) of collagens in *C. compactum*. Attenuated total reflection (ATR)–Fourier–transform infrared spectroscopy (FTIR) spectra reveal the collagen bands in both soluble (**A**) and insoluble (**B**) organic matrix fractions. [Reproduced from Rahman and Halfar (9)].

Recently, our group explored collagen in coralline red algae [5,9]. The research is now continuing, and a high number of collagens have now been extracted from this organism (papers in preparation). Some portions of this organism contain both chitin and collagen (Figure 4 and Figure 5). Because of the huge number of these organisms available in shallow water of the sea, it would be an easy way to collect this marine group for extracting collagen. However, purification of collagen from these organisms has been a problem, and this issue has already been mostly solved (see Section 3 for details). This is a new group of marine organisms, which could get special attention for the extraction of collagen molecules in the near future.

It is assumed that half of all marine-derived biomaterials are sourced from marine sponges, which might be the highest number of organisms in the invertebrates currently being used for the extraction of collagen. In sponges, collagen fibers have an interesting structural feature [45,49], and the molecules isolated from this group have a wide range of activities that can be used for promising biomedical applications [4,6], especially collagenous marine sponge skeletons, which are extremely strong, highly absorbent, elastic, and resistant to bacterial attack. A recent review by Ehrlich et al. [6] described details about collagen and collagen-like structural proteins from sponges. They also highlighted the prospects and trends of collagen extracted from sponges in biomedical applications, materials science, and technology. From the same research group [1], a hydroxylated fibrillar collagen containing an unusual motif of “Gly–3Hyp–4Hyp” was isolated from the glass sponge (Hexactinellida). The authors hypothesized that this motif in fibrillar collagen subject is a silica precipitation and a template for biosilicification. Recently, Tziveleka et al. [4] isolated and characterized the collagens from the marine demosponges *Suberites carnosus* (Suberitidae) and *Axinella cannabina* (Axenillidae) and found three different collagen-insoluble collagen (InSC), spongin-like collagen (SlC), and intracellular collagen (ICC) for biomedical applications. Collagen was isolated from many other marine sponges, for instance, *Chondrosia reniformis* [47], *Microciona prolifera*, *Spongia graminea*, *Haliclona oculata* [42], *Cacospongia scalaris*, *Hippospongia communis* [49], *Chondrosia reniformis* [50,51], *Geodia cydonium* [52], and several *Ircinia species* [53]. 

Corals are an abundant source of biologically and structurally active compounds. Coral skeletons have interconnected pores and are composed of CaCO_3_, with appropriate porosity and pore sizes, making them a suitable material for bone implant application [17]. Regarding its interesting structural formation, coral has been in use commercially since the 1990s and is available as interpore and bio-coral [21]. There are several studies that have been found for such kinds of application, e.g., a three-dimensional coral skeleton structure endorsed the hard tissue growth and was totally replaced by new bone [22]. Similarly, a coral skeleton was used in human grafting [23]. Because of the structural compositions of coral, it absorbed CaCO_3_ very quickly in growing new bone tissue, allowing for a formation of a scaffold. These reports indicate that the corals might have collagenous molecules, which can be applied as a treatment for bone-related disease. 

However, the research for collagen on corals, especially for soft corals, has remarkably improved. Over the last several years [2,3,44,54], very interesting findings on collagen molecules have been demonstrated from soft corals. Also, a number of collagen-associated glycoproteins have been detected in soft corals [14,24,25,55,56]. The researchers found unique collagen fibers from the soft coral *Sarcophyton ehrenbergi* [2,3]. These fibers expose a 3D structure and hyper-elastic behavior, which are analogous to natural human tissues. The peculiarity of these fibers is too long (9 ± 0.37 μm). The research also demonstrated the collagen I and II types. The structural characterization of these collagen fibers reveals a highly suitable biomaterial for medical applications. Benayahu et al. [54] invented an interesting patent from the same soft coral species *S. ehrenbergi*. The inventors claimed that “(1) the collagen fibers from the soft coral have high adjustable extensibility compared with mammalian collagen fibers and (2) the stiffness of the collagen fibers isolated from this species is at the top range of the reported stiffness range of mammal collagen fibers”. Another study [24] demonstrated the structural differentiation of collagen production in the ECM of soft corals; however, the authors did not investigate the types of collagen. Besides the above-mentioned marine organisms, sea urchin, marine fish, and mollusk organisms have been used for extracting different types of collagen, and the evidence showed that these collagen molecules have a strong role in the treatment of bone-related disease [20,27,29,30,31,33].

As mentioned above, research on the medical application of marine invertebrate collagen is currently progressing well. However, most collagen research findings from marine calcifiers/invertebrates are used in the application of bone-related disease (34), but the research in this field is still suffering from various complications. The medical application of marine collagens has been highlighted in recent reviews [6,31,35,57,58]. A review report by Cicciù et al. [57] suggested the facial bone reconstruction defect by applying marine collagen. During this review, the authors conducted a search using the MEDLINE and EMBASE databases (2007 to 2017), and their search results suggested that marine collagen can support the stability of the bone graft and could be an excellent carrier for growth factors. There are some recent reports of marine sources (coral, sponge, sea urchin, and fish) focused on the medical application (including bone tissue engineering and related diseases) of collagen available in the literature [3,6,17,31,59,60,61]. Moreover, collagen derived from mollusks, echinoderms, and sponges was reported [62,63,64,65,66,67,68,69,70,71], with some other important medical applications.

## 3. Purification Technique of Collagens from Marine Invertebrates 

The molecules in invertebrates are complex, and therefore the purification of any specific molecule from this group is tricky. An individual species is required to apply different techniques, as the characterization of their components is multifaceted. For instance, soft corals have sclerites and soft tissue (unlike the stony corals) comprising complex organic matrices [14]. For these complexities, it was difficult to purify molecules; however, our group successfully purified the molecules [11,14], including the functional extracellular matrix proteins (e.g., ECMP-67), enzymes, calcium-binding proteins, and glycoproteins (see Figure 3 for examples). Glycoprotein in the extracellular matrix protein is a key component of collagen (Figure 2) that plays the main role in the biological process of collagen in invertebrates. Applying similar techniques, we recently investigated coralline algae, which have a high concentration of both chitin and collagen biopolymers and are functional in both soluble and insoluble organic matrix fractions (Figure 4 and Figure 5) [5,9].

Coralline algal concentrations of the soluble organic matrix (0.9%) and insoluble organic matrix (4.5%) fractions are significantly higher than those of other marine invertebrates such as soft corals, with a soluble organic matrix and insoluble organic matrix of 0.03% and 0.05%, respectively [56,72]. The evidence of purified collagen in the coralline skeletons was also shown by X-ray diffraction (XRD) analysis (Figure 4). Jiang et al. [73] identified mineral crystals in collagen fibrils in a different marine invertebrate. The findings by Jiang et al. support our XRD results, and this technique has been revealed as a promising tool in analyzing collagens in the mineralization process. The results obtained by XRD demonstrated that XRD will become an important tool to study biological materials like collagen from the ECM of invertebrates. Such a high concentration of collagen present in the organic matrices of marine calcifiers presents the opportunity for future drug development in bone-related disease, and, moreover, both chitin and collagen present in the same species can take a significant role in drug design of other related diseases, because these two polymers are commonly used in drug design [74,75,76,77,78,79,80,81,82,83]. 

At present, the methods for the isolation and purification of collagens from the octocorals have been significantly improved. A patented protocol on the collagen purification from the soft coral [54] is now on the market. Since this method is patented, it is not open to the public. However, there are several publications by the same research group that currently exist in the literature, in which they established the methods in purifying collagens (including collagen types I and II) from the soft coral [2,3]. The development of these new technologies, along with the technologies established by our group as mentioned above, will be extremely beneficial for purifying functional collagens from these marine organisms. 

Despite the importance of collagenous marine sponge skeletons being documented, the techniques for the purification of collagens from this group are not well-established yet because of their insolubility and mineralization, which might cause difficulties in its separation and characterization [84,85]. However, researchers are trying to resolve these issues, and numerous investigations have so far been reported in this group [47,50,53,86,87]. Recently, Pozzolini et al. [47] established several new methods to purify collagenous fibrillar suspensions from the *Chondrosia reniformis* demosponge. The authors demonstrated that the obtained fibrillar collagens are extensively useful for tissue engineering and regenerative medicine, as well as in antioxidant activity. 

There are some techniques that have been established in purifying collagens from the invertebrates; however, a proteomic approach might be a useful tool to learn more about the collagen and its functions in detail. Proteomics have already been established as an important tool for the detection, characterization, and analysis of pharmaceutically useful proteins from marine organisms, and this approach provides the most precise evaluation of protein identities, abundance, composition, and protein expression profiling [5,26,88,89,90]. Therefore, in regard to collagen, the proteomics approach could be a promising toolkit in the near future. The overview regarding marine collagen of invertebrates stated above allows us to understand some newly developed techniques and suitable methods for extracting and purifying collagen, as well as for applying proteomics approach for medical applications.

## 4. Future Applications of Invertebrate Collagens in Medical Field

The marine ecosystem provides suitable and numerous diversified resources for human health in comparison to the terrestrial ecosystem. In the last few decades, marine resources, especially invertebrates, have been recognized to be a promising source for many drugs (e.g., Cytarabine, Vidarabine, and Halichondrin B) [91]. According to the discussion above (Section 1, Section 2 and Section 3), marine invertebrates and related calcifying organisms such as soft and hard corals, sponge, mollusk, sea urchin, and coralline algae could be a major source of medicines over the next decades. However, extraction and purification of collagen for the purpose of medical application of these resources is still under investigation developing. Despite some impressive work having been performed on collagenous sponges and corals [1,2,3,4,6,7,15,16,17,47,54,91,92,93], an intensive study is necessary with these two groups and other invertebrates to use these huge apposite resources in future years. The potential of marine invertebrates for collagen could be realized by developing new technologies; indeed, there are many methods such as proteomics, computer-aided design, bioinformatics, and combinatorial synthesis that are now being applied. 

The biological diversity of marine invertebrates and complex protein and peptide components direct us toward discovery of many new drugs for various therapeutic areas, including bone-related disease (e.g., osteoporosis) [94]. Besides cancer, microbial infections, and inflammation, drug discovery for bone-related disease is the biggest challenge of the current century, and collagen extraction from marine invertebrates shows new promise in fighting against this and other related diseases.

## 5. Concluding Remarks

In this review, the current state of research on collagen extracted from the ECM of invertebrates and its applications in the medical field have been discussed, and some light has been shed on future perspectives of this important marine material. The methodological issues of collagen purification from invertebrates, which the researchers are currently struggling with, have also been highlighted. The discussion concerning the purification techniques in this review could be of tremendous help in the extraction of purified collagen from invertebrates. The extracellular matrix, which is one of the key components in invertebrates and is responsible for producing collagen in this marine group, has been elaborated with informative imaging. In addition, the glycosylation activity with the formation of glycoproteins (size of the protein, which varies from species to species) in invertebrates, whose biological processes are involved in producing collagen, has been discussed for the first time in this review. The obtained results demonstrate the potential for marine invertebrates to generate new drugs, especially for bone tissue regeneration.
